# Dielectric Jump and Negative Electrostriction in Metallosupramolecular Ionic Crystals

**DOI:** 10.1038/s41598-018-20750-1

**Published:** 2018-02-08

**Authors:** Satoshi Yamashita, Yasuhiro Nakazawa, Shusuke Yamanaka, Mitsutaka Okumura, Tatsuhiro Kojima, Nobuto Yoshinari, Takumi Konno

**Affiliations:** Department of Chemistry, Graduate School of Science, Osaka University, Machinakeyama 1-1, Toyonaka, Osaka, 560-0043 Japan

## Abstract

In natural ionic solids, cationic and anionic species are alternately arranged to minimize electrostatic energy. Aggregation of identical ionic species is commonly prohibited due to the repulsive, long-range nature of Coulombic interactions. Recently, we synthesized unique ionic solids, [Au^I^_4_Co^III^_2_(dppe)_2_(d-pen)_4_]X_2_·*n*H_2_O (dppe = 1,2-bis(diphenylphosphino)ethane, d-pen = d-penicillaminate), in which complex cations are self-assembled into a cationic supramolecular octahedron, while monovalent or divalent inorganic anions are aggregated into an anomalous anionic cluster accommodating several water molecules. This quite unusual aggregation manner originates from various molecular-level non-Coulombic interactions such as hydrogen bonds and CH-π interactions; thus, this class of ionic solids is referred to as non-Coulombic ionic solids, abbreviated as NCISs. Herein, we report that the NCISs with a peculiar charge-separated (CS) structure in a cubic lattice show a negative, isotropic electrostriction phenomenon that has never been found in any ionic solids, as well as an anomalously large relaxer-like dielectric jump phenomenon reaching to an application level of *ε*′/*ε*_0_ ~ 10^5^. The appearance of these phenomena was explained by the cooperative dynamics of inorganic anions and dipolar water molecules in the pliable anionic clusters that are surrounded by a rather robust cationic metallosupramolecular framework with a meso-scopic scale.

## Introduction

**Figure 1:**
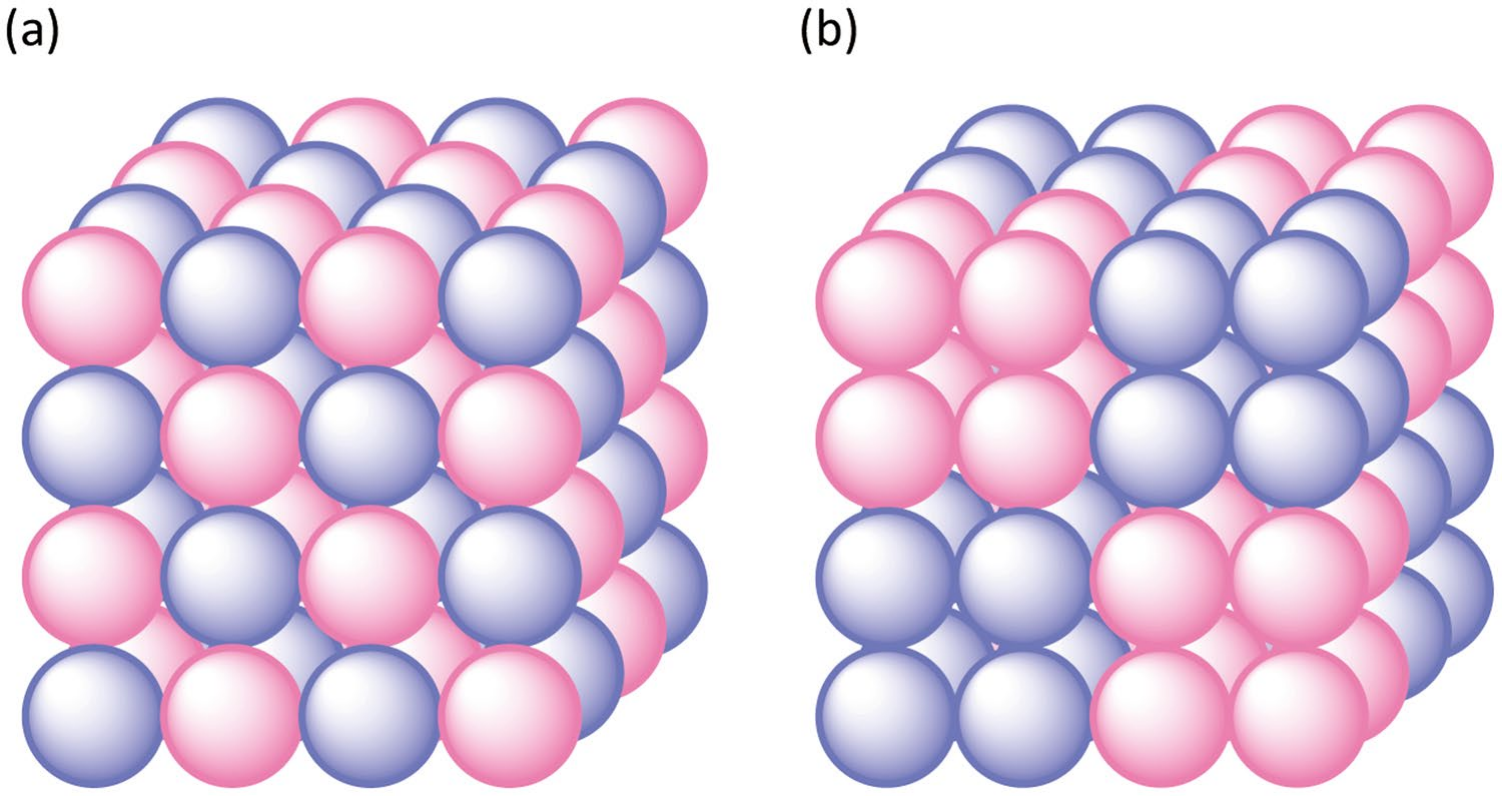
(**a**) Schematic illustration of (**a**) common and (**b**) charge-separation type non-Coulombic ionic solid (CS-NCIS) materials. Red and blue balls represent cationic and anionic species.

It has been recognized that a variety of polynuclear and supramolecular coordination compounds are created from thiol-containing amino acids, such as l-cysteine (l-H_2_cys), d-penicillamine (d-H_2_pen), and their derivatives, in combination with 3d and 4d transition metal ions, because these amino acids can adopt various coordination modes to multiple metal centres by using amine, carboxyl, and/or thiol groups dependent on their protonation/deprotonation states^1–4^. We found that the introduction of hydrophobic dppe (1,2-bis(diphenylphosphino)ethane) into a gold(I)-cobalt(III) coordination system with hydrophilic d-pen affords a cationic Au^I^_4_Co^III^_2_ hexanuclear complex, [Au_4_Co_2_(dppe)_2_(d-pen)_4_]^2+^ ([**1**]^2+^), in which two Co^III^ centres are spanned by two [Au^I^_2_(dppe)(d-pen)_2_]^2−^ linkers^1–3^. On crystallization with appropriate monovalent inorganic anions, such as ClO_4_^−^, NO_3_^−^, and Cl^−^ or divalent anions such as SiF_6_^2−^ and SO_4_^2−^, the 6 Au^I^_4_Co^III^_2_ cations of [**1**]^2+^ are self-assembled to form huge cationic supramolecular octahedrons, {[**1**]^2+^}_6_, which are closely packed into a face centred cubic (fcc) structure with a lattice parameter of *a ≈* 3.8 nm. The mesoscopic-scale fcc structure involves large hydrophilic and hydrophobic tetrahedral interstitial spaces with volumes of ~900 Å^3^ and ~1500 Å^3^, respectively, besides small octahedral interstitial spaces with a volume of ~70 Å^3^. In each hydrophilic tetrahedral interstice, 10 monovalent anions or 6 divalent anions are aggregated in adamantane-like {X^−^}_10_ or octahedron-like {X^2−^}_6_ cluster structures, together with several water molecules. On the other hand, each hydrophobic tetrahedral interstice is occupied by a number of water molecules to form a huge water cluster, which works as a reservoir of water molecules in the crystal. As a consequence of the delicate balance of condensation energies, the cationic supramolecular octahedrons and the inorganic anion clusters are alternately arranged in the crystal, constructing a giant zinc-blend-like lattice structure with a large charge-separation state. In this class of ionic solids, which is referred to as charge-separation type non-Coulombic ionic solids (CS-NCISs) (Fig. 1), the cationic framework consisting of the supramolecular octahedrons is rather robust even at a high temperature, whereas the portion of the anionic clusters composed of inorganic anions and water molecules appears to be quite flexible. Such being the case, the deformation of the local structure by external stimuli, especially by electric fields, possibly induces unexpected changes in the dielectric and elastic properties through the coupling of charge and lattice degrees of freedom with retaining the framework. Throughout systematic experiments and analyses using single crystals of the CS-NCISs, we found not only an extraordinary large relaxer-like dielectric jump in a symmetric cubic crystal system but also an unprecedented lattice contraction upon the application of electric fields. Herein, we wish to report these remarkable discoveries that have never been found in metal-organic ionic solids.

Previously, the crystal structures of [**1**]X_2_·*n*H_2_O [X_2_ = Cl_2_, Br_2_, (NO_3_)_2_, (ClO_4_)_2_, SO_4_, SiF_6_] were investigated using a laboratory X-ray source at 200 K^2,3^. However, the positions of the water molecules were not clearly determined owing to the limited data quality and their disordered structure. That is, for [**1**](ClO_4_)_2_·*n*H_2_O and [**1**](NO_3_)_2_·*n*H_2_O, no water molecules were found in the anionic cluster that existed in each hydrophilic tetrahedral interstice, although several water molecules were observed for [**1**]Cl_2_·*n*H_2_O, [**1**]Br_2_·*n*H_2_O, [**1**](SO_4_)·*n*H_2_O, and [**1**](SiF_6_)·*n*H_2_O. In addition, water molecules that existed in the hydrophilic tetrahedral interstices could not be modelled for all [**1**]X_2_·*n*H_2_O. In this work, the crystal data of [**1**]X_2_·*n*H_2_O were collected using synchrotron X-ray radiation at 100 K to determine the positions of the water molecules and inorganic anions more precisely.

**Figure 2:**
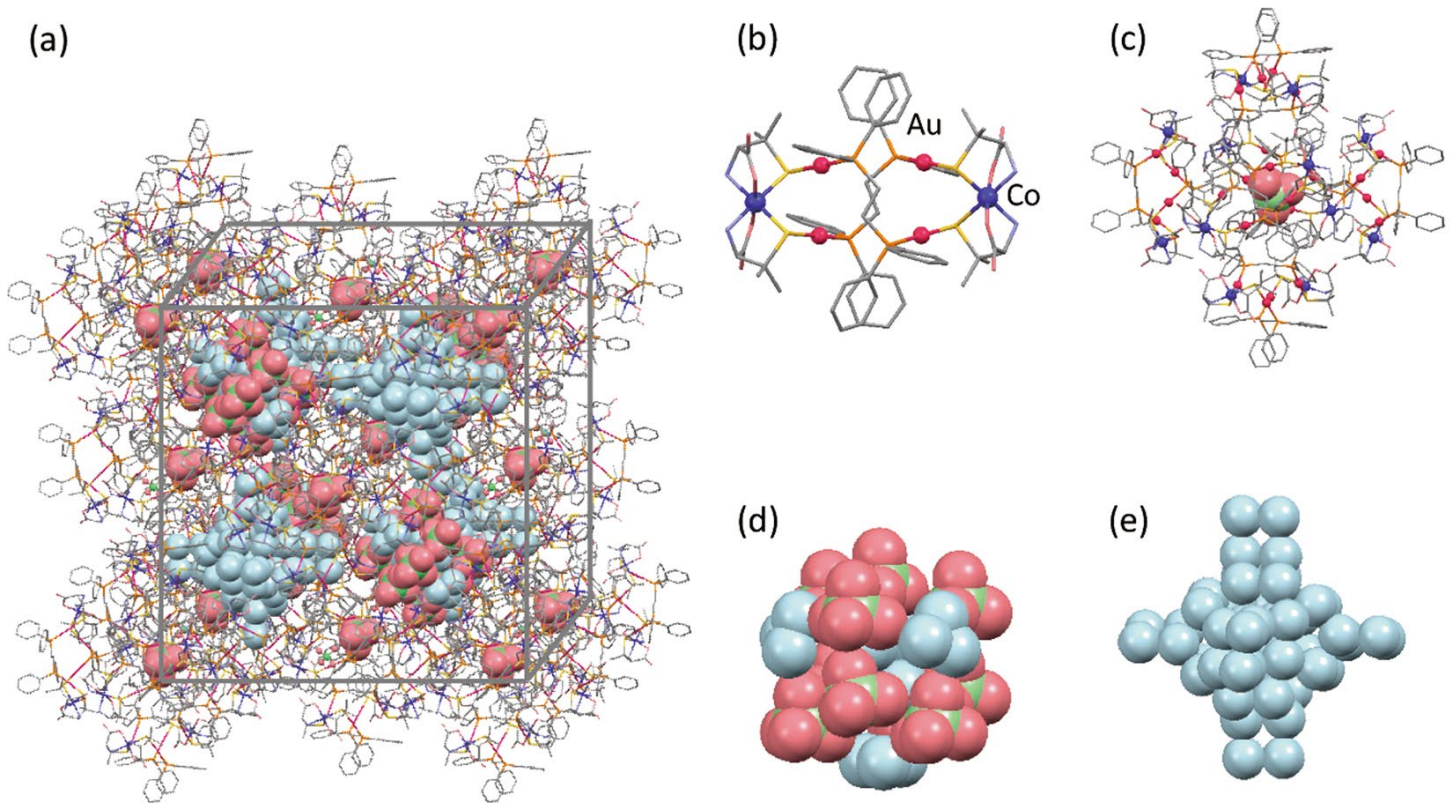
Structures of (**a**) packing, (**b**) complex-cation, (**c**) cationic supramolecular octahedron, (**d**) inorganic-anion cluster, and (**e**) water cluster in [**1**](ClO_4_)_2_∙*n*H_2_O.

The synchrotron single-crystal X-ray analysis of [**1**](ClO_4_)_2_·*n*H_2_O clearly indicated the presence of 2 water molecules that are disordered inside the adamantane-like {ClO_4_^−^}_10_ cluster, although the overall crystal structure is essentially the same as that determined previously (Fig. 2). Moreover, 4 water molecules that are also disordered were found on the surface of the {ClO_4_^−^}_10_ cluster. These water molecules appear to moderate the Coulombic repulsion between ClO_4_^−^ ions in the cluster. This is also the case for [**1**](NO_3_)_2_·*n*H_2_O; two water molecules that are disordered were found inside the adamantane-like {NO_3_^−^}_10_ cluster (Fig. S1). In this case, however, 4 of 10 nitrate anions, which are located at the tetrahedral site of the adamantane, are disordered over two positions. This disorder makes it difficult to determine the positions of water molecules located on the surface of the {NO_3_^−^}_10_ cluster. The numbers and the positions of water molecules inside and on the surface of the inorganic-anion cluster were precisely determined for the other [**1**]X_2_·*n*H_2_O; [**1**]Cl_2_·*n*H_2_O and [**1**]Br_2_·*n*H_2_O each accommodate 4 water molecules inside the adamantane-like {X^−^}_10_ cluster and 12 water molecules on the surface of the {X^−^}_10_ cluster, whereas [**1**](SO_4_)·*n*H_2_O and [**1**](SiF_6_)·*n*H_2_O each accommodate 4 water molecules inside the octahedron-like {X^2−^}_6_ cluster and 4 water molecules on the surface of the {X^2−^}_6_ cluster (Fig. S1). In addition, a large water cluster that consists of more than 36 water molecules, some of which are disordered, was commonly found for all [**1**]X_2_·*n*H_2_O in each hydrophobic tetrahedral interstice (Figs 2 and S1). The synchrotron X-ray analysis was also carried out at a higher temperature for [**1**](NO_3_)_2_·*n*D_2_O, which demonstrated the thorough retention of its crystallinity even at 370 K (Fig. S2). The molecular structure of [**1**]^2+^ and its special arrangement in the crystal at 370 K are essentially the same as those at 100 K. However, a slight contraction of the {NO_3_^−^}_10_ cluster, together with the directional change of each NO_3_^−^ anion, was observed by increasing the temperature; the average N … N distances are 4.72 Å and 4.63 Å at 100 K and 370 K, respectively. In addition, most of the water molecules were not found from the difference Fourier map at 370 K, presumably due to the severe dynamic disorder of water molecules in the crystal. These observations suggest a high motility of inorganic anions and water molecules accommodated in the rigid supramolecular framework composed of [**1**]^2+^.

**Figure 3:**
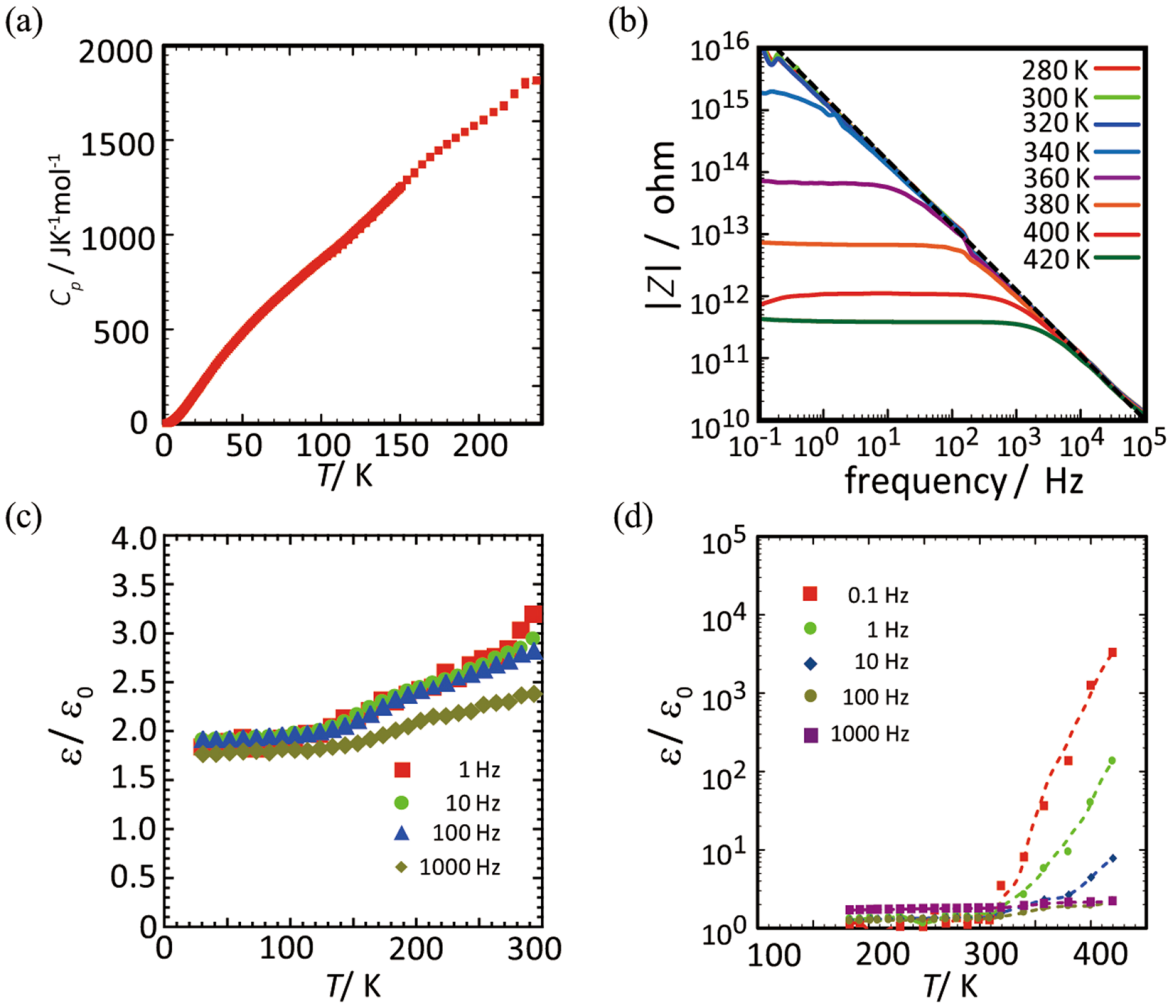
(**a**) Temperature dependence of the heat capacity of [**1**](ClO_4_)_2_∙*n*H_2_O. (**b**) Temperature and frequency dependences of the absolute value of the complex ac impedance of [**1**](ClO_4_)_2_∙*n*H_2_O. The dashed lines in the figure show a para-electric state corresponding to a dielectric constant (*ε*′/*ε*_0_) of 1.5–3.0. (**c**) The temperature dependence of the real part of *ε*′/*ε*_0_ for [**1**](ClO_4_)_2_∙*n*H_2_O between 30 K and 300 K. (**d**) The temperature dependence of the real part of *ε*′/*ε*_0_ for [**1**](ClO_4_)_2_∙*n*H_2_O between 180 K and 420 K in logarithmic plot.

To gain more insight into the thermal stability of the CS-type NCISs, the temperature dependency of the heat capacity (*C*_*p*_) was measured for [**1**](ClO_4_)_2_∙*n*H_2_O (Fig. 3a). There are no significant thermal anomalies in the temperature range of 2.0 K–240 K. This clearly indicates that the structure with the cubic space group of *F*23 is stable in this temperature range; the structural deformation to monoclinic or triclinic phases does not occur despite the drastic charge-separation state of [**1**](ClO_4_)_2_∙*n*H_2_O in the crystal. The crystal stability up to 450 K was also confirmed by the differential thermal analysis (DTA); no structural phase transition and decomposition peaks were observed up to this temperature, although the release of water molecules was detected by the thermogravimetry analysis (TGA) above 380 K (Fig. S3). The release of water molecules was prohibited when the crystal surface was coated by an epoxy resin. Note that the *C*_*p*_ values increase monotonously with the temperature, even above 200 K (Fig. 3a). This implies that the heat capacity does not obey the typical Debye feature presumably because of the significant contribution of optical phonon modes that originate from the translational and rotational motions of small molecules (H_2_O and/or inorganic anions) in the interstitial spaces with increasing temperature.

**Figure 4:**
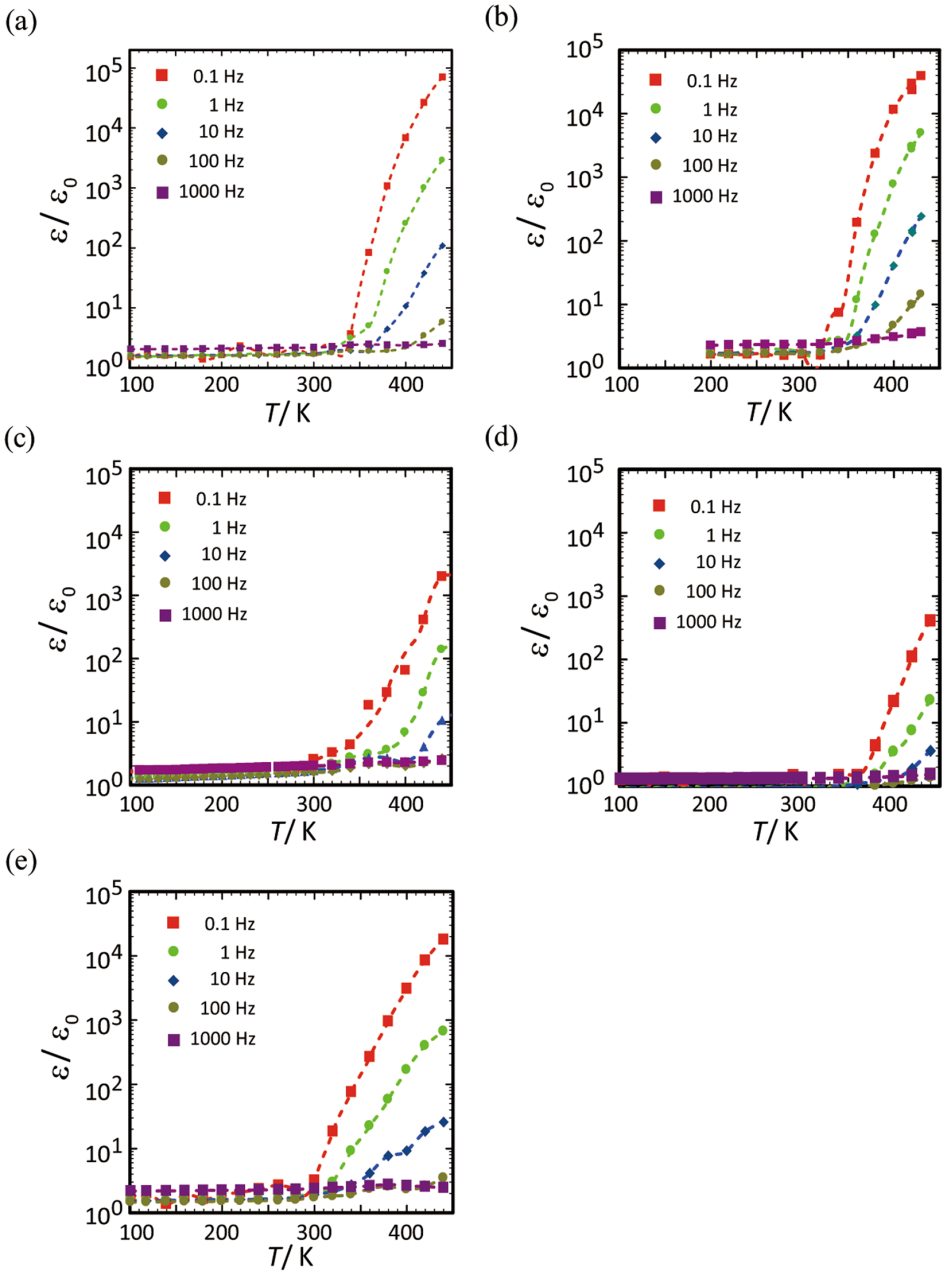
Temperature dependencies of *ε*′/*ε*_0_ for (**a**) [**1**]Cl_2_∙*n*H_2_O, (**b**) [**1**]Br_2_∙*n*H_2_O, (**c**) [**1**](NO_3_)_2_∙*n*H_2_O, (**d**) [**1**](SO_4_)∙*n*H_2_O, and (**e**) [**1**](SiF_6_)∙*n*H_2_O.

Figure 3b shows the frequency (*ω*) dependency of the absolute values of the ac impedance, *Z*, in the temperature range from 280 K to 420 K for a single-crystal of [**1**](ClO_4_)_2_∙*n*H_2_O. In the low temperature region, log|*Z*| obeys a linear relation with log*ω* in the wide frequency region between 10^–1^ Hz and 10^5^ Hz. This means that the capacitance values are nearly constant. Moreover, the real part of the relative permittivity (*ε*_r_ = *ε*′/*ε*_o_) is within a range of 1.5–3.0, which is on the same order as insulating materials, such as SiO_2_ glasses, quartz, and sapphire (Fig. 3c,d)^5,6^. However, the relative permittivity changes abruptly just above room temperature with a kink feature in log|*Z|* (Fig. 3b). While the deformation of crystal symmetry with increasing temperature has generally been observed for ferroelectric transition metal oxides such as BaTiO_3_ (BTO)^6^ and K(NH_4_)_2_PO_4_ (KDP)^7^ and organic charge-transfer complexes such as TTF-CA^8,9^, no deformation was noticed for [**1**](ClO_4_)_2_∙*n*H_2_O. The abrupt increase of the *ε*′/*ε*_0_ values is commonly observed for the other CS-NCISs having different anionic clusters, [**1**]X_2_∙*n*H_2_O (X_2_ = (NO_3_)_2_, Cl_2_, Br_2_, SO_4_, SiF_6_), although the temperature and magnitude are dependent on the type of anion included (Fig. 4). The *ε*′/*ε*_0_ values at 10^−1^ Hz for these compounds reach to 10^3^–10^5^, except for [**1**](SO_4_)∙*n*H_2_O; these values are in the highest class among those found in various molecule-based dielectric compounds and are comparable with those reported in the relaxer systems of some functional ceramics^9,10^. The observed temperature dependency of *ε*′/*ε*_0_ indicates that this permittivity jump is dominated by an activation-type of dynamics. The evaluation of the onset temperature of the permittivity jump at various frequencies revealed the nearly linear relation in log*ω* vs. 1/*T*_onset_. From this relation, the activation energies are estimated to be 50–100 kJ mol^−1^ (Fig. S4), which are on a similar order to those found in relaxers such as BaTiO_3_ (BTO) and Pb(Mg_1−*x*_Nb_*x*_)O_3_ (PMN)-based compounds^10,11^. Note that the gradual increase of *ε*′/*ε*_0_ for [**1**](ClO_4_)_2_∙*n*H_2_O starts from 150–160 K as a precursor of the abrupt jump (Fig. 3c). Since this temperature range is close to the glass transition temperature of supercooled water embedded in nano-porous or polymer systems^12–15^, the dielectric behaviour for [**1**](ClO_4_)_2_∙*n*H_2_O is most likely due to the molecular dynamics triggered by dipolar water molecules existing in the anionic cluster of {ClO_4_^−^}_10_. It has been recognized that water molecules confined in nano-pores can form a supercooled liquid state to give a rather large dielectric response^16,17^. However, such dynamics in a metastable liquid state has been realized only in wet conditions obtained by exposing framework materials to water. Thus, the dielectric dynamics realized for the CS-NCISs ([**1**]X_2_∙*n*H_2_O) under non-wet conditions is a significant aspect of the phenomena to be emphasized.

**Figure 5:**
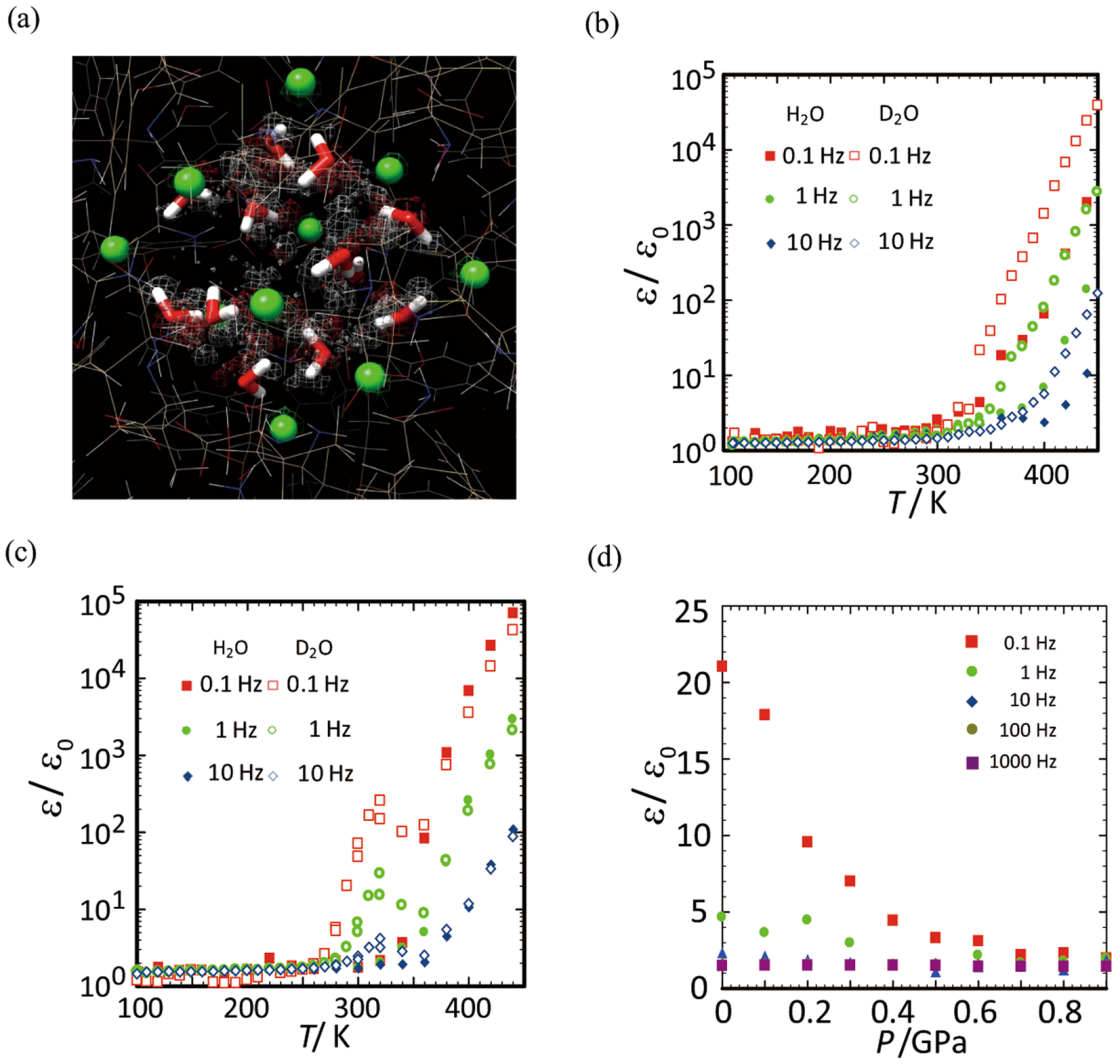
(**a**) Probability distributions of the inorganic-anion cluster in [**1**]Cl_2_∙*n*H_2_O, obtained from QM/MM MD calculations at 300 K. The colours of the wire meshes for Cl, O, and H atoms are green, red, and white, respectively. The X-ray structure is also shown in the sphere (Cl^−^) and the stick (H_2_O) models. (**b**) Temperature dependencies of *ε*′/*ε*_0_ for [**1**](NO_3_)_2_∙*n*H_2_O (closed symbol) and [**1**](NO_3_)_2_∙*n*D_2_O (open symbol) at 0.1 Hz, 1.0 Hz, and 10 Hz. (**c**) Temperature dependencies of *ε*′/*ε*_0_ for [**1**]Cl_2_∙*n*H_2_O (closed symbol) and [**1**]Cl_2_∙*n*D_2_O (open symbol) at 0.1 Hz, 1.0 Hz, and 10 Hz. (**d**) External pressure dependencies of *ε*′/*ε*_0_ for [**1**]Cl_2_∙*n*H_2_O at 350 K.

To inspect the dynamic behaviour of the anionic clusters accommodating water molecules, we performed molecular dynamics (MD) calculations with a quantum mechanics (QM)/molecular mechanics (MM) model for [**1**]Cl_2_∙*n*H_2_O at 300 K. Figure 5a illustrates the equivalued surfaces of the probability densities for Cl^−^ anions and O and H atoms (*ρ*(X) = 0.005, X = Cl^−^, O, H) as wire meshes. The X-ray structure positions for Cl^−^ anions, water molecules, and other molecules are also shown in the sphere, stick, and wire models, respectively. From this figure, it is seen that the positions of Cl^−^ anions are rather stable at room temperature, while the water molecules fluctuate considerably but remain bound to the anionic cluster to moderate the repulsive interaction between Cl^−^ anions. This situation allows them to have a liquid-like feature at approximately room temperature. Therefore, it is assumed that the frequency-dependent motion of dipolar water molecules induced by ac electric fields is a trigger for the dielectric jump, as in the case of water molecules in nano-porous systems^18,19^. The calculations also supported that the liquid-like dynamics of water molecules in the cluster can induce the structural distortion of the whole anionic cluster. This cooperative effect accounts for the unexpectedly large dielectric jump phenomenon in the CS-NCISs of [**1**]X_2_∙*n*H_2_O. In addition, the anion dependency on the dielectric features is understood by the difference in the rigidity of water molecules accommodated in the anionic cluster. That is, [**1**]Cl_2_∙*n*H_2_O that contains the anion clusters of {Cl^−^}_10_ with a sparse structure shows the most sensitive relaxer feature, whereas [**1**](ClO_4_)_2_∙*n*H_2_O that has the anion clusters of {ClO_4_^−^}_10_ with a dense structure requires a substantially higher temperature to induce a relaxer feature. It is worth mentioning that the cooperative dynamics is also crucial to the appearance of the quite large *ε*′/*ε*_0_ values exceeding 10^5^.

The presence of the cooperative dynamics due to inorganic anions and water molecules in the anionic cluster is also supported by the appearance of isotope and external pressure effects on the dielectric behaviour. As shown in Fig. 5b, the *ε*′/*ε*_0_ values of the deuterated [**1**](NO_3_)_2_∙*n*D_2_O are enhanced by nearly one order compared with those of the original sample of [**1**](NO_3_)_2_∙*n*H_2_O. A similar tendency was observed for the deuterated [**1**](SO_4_)∙*n*D_2_O and [**1**](SiF_6_)∙*n*D_2_O (Fig. S5). While [**1**]Cl_2_∙*n*D_2_O does not show a drastic enhancement (Fig. 5c), the onset temperature is lower than that of [**1**]Cl_2_∙*n*H_2_O, suggesting a weak interaction of water molecules with Cl^−^ anions compared with the interaction with NO_3_^−^ anions. These observations demonstrate that the dynamics of water molecules in [**1**]X_2_∙*n*H_2_O is enhanced by the deuteration. Furthermore, the positive isotope effect observed here supports the importance of the dynamic features of water molecules to the appearance of the permittivity jump, considering that the weakening of hydrogen-bonding interactions in the cluster by the deuteration results in the increase of water molecule motion. Figure 5d shows the pressure and frequency dependences of *ε*′/*ε*_0_ for [**1**]Cl_2_∙*n*H_2_O at 350 K. The dielectric permittivity observed at ambient pressure is suppressed drastically with increasing pressure, and the increase of the dielectric permittivity is nearly completely suppressed above 0.7 GPa. It is reasonable to assume that the increase of external pressures causes the contraction of the anionic cluster space, preventing the dynamics of water molecules and inorganic anions needed for the appearance of a large dielectric permittivity.

**Figure 6:**
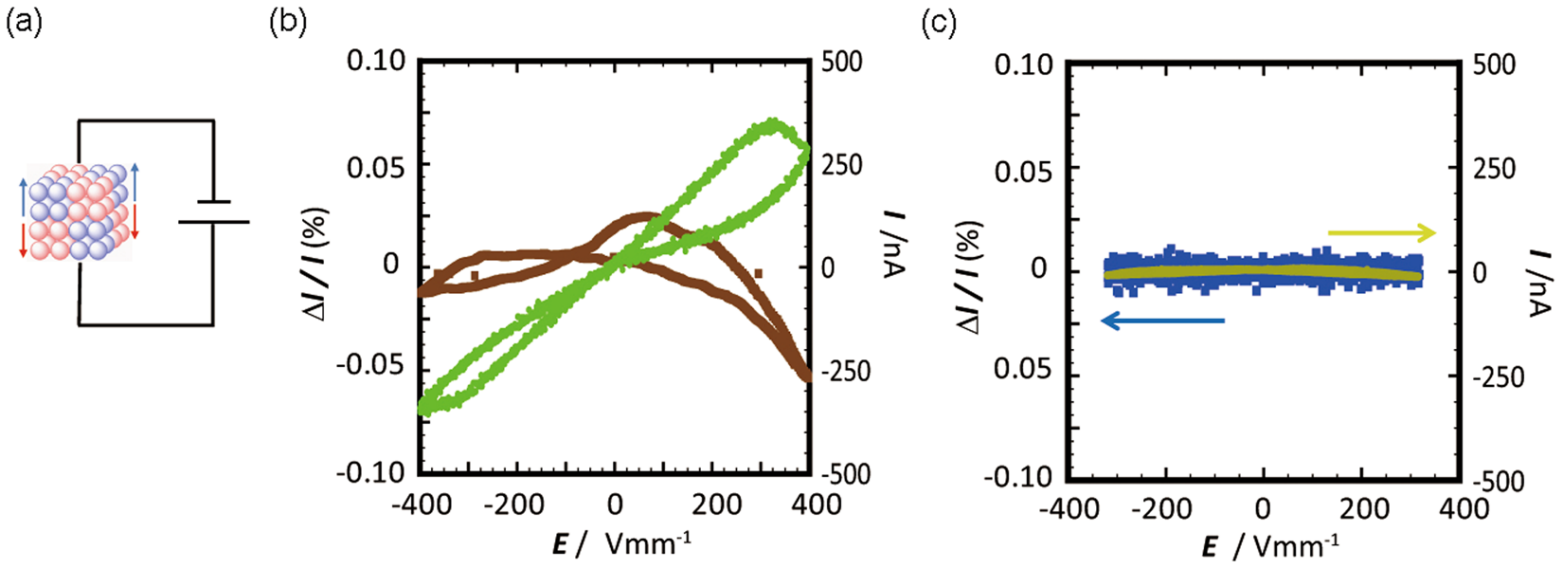
(**a**) Schematic illustration of the electrostriction effect. Simultaneous measurements of the *I-V* characteristics and the displacement of the sample position from the AFM tip at (**b**) 380 K and (**c**) room temperature.

We expected that the peculiar charge-separation state in the CS-NCISs of [**1**]X_2_∙*n*H_2_O consisting of the cationic supramolecular octahedrons of {[**1**]^2+^}_6_ and the anionic clusters of {X^−^}_10_ or {X^2−^}_6_ leads to the appearance of unusual charge-lattice coupling phenomena. Since the total charge valences of {[**1**]^2+^}_6_ and {X^−^}_10_ or {X^2−^}_6_ are significantly larger than those of the cationic and anionic species in normal ionic solids^18^, the application of a DC voltage seems to induce some strain in the crystal because of the opposite displacement of plus- and minus-charged cluster units by the electric field (Fig. 6a). To check this electrostriction effect, we carried out *Q-V* and *I-V* measurements for [**1**]Cl_2_∙*n*H_2_O (Fig. 6b,c), which shows the largest dielectric jump among [**1**]X_2_∙*n*H_2_O (Fig. 4). The crystal contraction/extension was also measured for [**1**]Cl_2_∙*n*H_2_O by using AFM tips, with a sweeping DC voltage of up to ±400 V mm^−1^ (Fig. 6b,c). The *I-V* characteristic measured at 380 K shows a butterfly-type hysteretic feature, which is not observed at room temperature. Since the increase of current means a charging because of the increase of polarization produced by the deformation of the crystal lattice^18,19^, the appearance of the hysteresis is attributed to an electrostriction. The electric contraction/extension was directly detected by using an AFM tip and simultaneous sweeping in the *I-V* measurements at 380 K, but not at room temperature (Fig. 6b). Thus, it is concluded that the electrostriction occurs synchronized with the electric charging in the crystal lattice at 380 K, while the changes in the polarization and displacement are not caused at room temperature. From the data in Fig. 6b, the relative value of the displacement, Δ*l*/*l*, was evaluated to be 0.07% at 400 V mm^−1^. This value is three times larger than that for PZT^18^ and is comparable with the value for BaTiO_3_, which was recently attained by the domain conversion technique^19^. More remarkably, the electrostriction effect occurs in the negative direction, that is, the crystal shrinkage occurs with increasing DC voltage applied to the crystal; although the charging feature is quite similar to that found in ceramics. Such an electrostriction with a negative displacement is unprecedented; normal electrostriction, as found in ceramics, is of positive displacement^18,19^. We also performed the crystal displacement measurements with increasing electric fields for a different, 10 times smaller sample. A similar, negative displacement feature was observed in both parallel and perpendicular directions (Fig. S6), although a slight difference was noticed between them. The crystal shrinkage with increasing DC voltage, which occurs in all *x-*, *y-*, and *z*-axes, was also recognized by the tracing of the Bragg spot positions and the refinement of the cell parameters in the single-crystal X-ray analysis of [**1**]Cl_2_∙*n*H_2_O (Fig. S7). These results imply that the total volume of crystalline [**1**]Cl_2_∙*n*H_2_O decreases with increasing voltage through the negative electrostriction. While the detailed mechanism of this negative electrostriction is not clear at present, we speculate that the removal of water molecules from the inorganic-anion clusters, which is induced by the strong striction and distortion by the electric field, leads to the shrinkage of the inorganic-anion clusters to contract the whole crystal that possesses a cubic lattice structure.

In summary, we succeeded in deriving remarkable dielectric features peculiar for CS-NCISs ([**1**]X_2_∙*n*H_2_O) and discussed their mechanisms in terms of the soft dynamics of the anion clusters ({X^−^}_10_ or {X^2−^}_6_) triggered by water molecules accommodated in the clusters. We also found the unprecedented negative electrostriction for [**1**]Cl_2_∙*n*H_2_O, which is induced by a DC bias application. These novel features for CS-NCISs should be explored for potential applications in sensing and/or vibration devices. The coexistence of robust cationic and soft anionic cluster regions in CS-NCIS is expected to lead to other novel functionalities based on the local non-equilibrium feature in a rigid crystal lattice, triggered by the activation of water molecules under non-wet conditions. We emphasize that NCISs might be a new generation of materials in which multiscale aspects coexist in the same crystal, as if in biological materials.

## Methods

The synthesis of [Au^I^_4_Co^III^_2_(dppe)_2_(d-pen)_4_]^2+^ and the growth of its single crystals with monovalent or divalent counteranions were performed according to the method reported in refs^2,3^. The diffraction data for [**1**]Cl_2_∙*n*H_2_O was recorded at 100 K with a RIGAKU/MSC Mercury CCD X-ray diffractometer with synchrotron radiation (λ = 0.6889 Å) at PF-AR (NW2A beamline) of the High Energy Accelerator Research Organization (KEK). The diffraction images were processed by using HKL2000. The diffraction data for [**1**](ClO_4_)_2_, [**1**](NO_3_)_2_, [**1**]Br_2_, [**1**]SO_4_ and [**1**]SiF_6_ were recorded at 100 K with an ADSC Q210 CCD area detector with synchrotron radiation (λ = 0.6500 Å for [**1**](NO_3_)_2_, [**1**]Br_2_ and [**1**]SiF_6_ and 0.7000 Å for [**1**](ClO_4_)_2_, and [**1**]SO_4_) at the 2D beamline at the Pohang Accelerator Laboratory (PAL). The intensity data were processed using the HKL3000 program, collected by using the *ω*-scan technique, and semi-empirically corrected for absorption by using PLATON. The diffraction data for [**1**](NO_3_)_2_·*n*D_2_O were recorded at 300 K, 343 K and 423 K with a RIGAKU/Mercury 2 CCD detector with synchrotron radiation (λ = 0.7003 Å) at Spring-8 (BL02B1 beamline). The diffraction images were processed by using RAPID-AUTO. The tracing experiments of Bragg spots by X-ray diffraction with increasing DC voltages were performed with a RIGAKU/MSC Mercury CCD X-ray diffractometer with synchrotron radiation (λ = 0.6889 Å) at PF-AR (NW2A beamline) of the High Energy Accelerator Research Organization (KEK). The diffraction images of the single crystal (0.25 × 0.25 × 0.15 mm^3^) of [**1**]Cl_2_ with two lead wires (the sample was coated with epoxy) were recorded at 380 K. The diffraction images shown in Fig. S7 were collected by using the *ω*-scan technique (∆*ω* = 3 °, exposure time = 3.0 sec) under both conditions without voltage and with 100 V. The cell parameters were determined after the refinements based on the 2321 for 0 V and 2124 for 100 V reflections in a *ω* range = 0–30 ° by using Crystal Clear 2.0. 0 V: Cubic *F*, *a* = 37.6209(54) Å, *V* = 53246 (11) Å^3^. 100 V: Cubic *F*, *a*′ = 37.5553(59) Å, *V*′ = 52968(12) Å^3^.

The temperature and frequency dependencies of the ac impedance for [**1**]Cl_2_∙*n*H_2_O were measured by the Solartorn1260 system with the dielectric measurement interface 1296. The frequency range of the system is from 10^−1^ Hz to 10^5^ Hz, and the temperature range is from 30 K to 440 K. The temperature of the sample part was controlled within ±0.1 K using the temperature controller LakeShore 335. Single crystals with a typical size of 0.4 × 0.4 × 0.2 mm^3^ were used for measurements by the four/two terminal method. A thin gold wire with a diameter of 30 μm was attached to a crystal using carbon paste (Fujikura DOTITE XC-12). To prevent the release of water of crystallization, the crystal surface was coated by an epoxy resin. The temperature dependence of the permittivity was calculated from the ac impedance using the formula of *Z*′′/*C*_0_*ωZ*^2^, where *Z*′′, *C*_0_, *ω* and *Z* are ideally part of the impedance, base capacitance of the set-up, frequency and measured impedance, respectively.

The polarization measurements with a DC electric voltage was performed on a FET-1E system (TOYO Cooperation) using the *Q-V*, *I-V* mode. A single crystal with two lead wires was coated by epoxy, to ensure the contact of the current leads on the crystal and to prevent the release of water molecules in the dry atmosphere. The simultaneous measurements of the polarization and displacement of the crystal by sweeping electric voltage were performed by the combined FET-1E with AFM measurements. The temperatures of the sample stage were controlled to within ±1.0 K using a chip-type Pt sensor.

The numerical studies were performed by quantum mechanics (QM)/molecular mechanics (MM) MD simulation methods. The simulation was performed with Amber14. The details of the model and calculation procedures are explained in the supporting information (Fig. S8). The validity of the liquid-like feature of water molecules was discussed through the comparison with the ab initio QM result (Figs S8, S9).

### Accession codes

The X-ray crystallographic coordinates for the structure reported in this Article have been deposited at the Cambridge Crystallographic Data Centre (CCDC) under deposition number CCDC 1578196–1578204. These data can be obtained free of charge from the Cambridge Crystallographic Data Centre via www.ccdc.cam.ac.uk/data_request/cif.

## Electronic supplementary material

Supplementary information
