# Supplementary material for: Dielectric Jump and Negative Electrostriction in Metallosupramolecular Ionic Crystals
Source: Sci Rep. 2018 Feb 8;8:2606. doi: 10.1038/s41598-018-20750-1 (PMC5805720; doi:10.1038/s41598-018-20750-1)
Supplement: Supplementary file 1 — Supplementary information [file 41598_2018_20750_MOESM1_ESM.pdf]

Supplemental Information

Dielectric Jump and Negative Electrostriction in Metallosupramolecular Ionic Crystals

Satoshi Yamashita, Yasuhiro Nakazawa*, Shusuke Yamanaka, Mitsutaka Okumura, Tatsuhiro Kojima, Nobuto Yoshinari, Takumi Konno*

Department of Chemistry, Graduate School of Science, Osaka University, Machinakeyama 1-1, Toyonaka, Osaka 560-0043, Japan

Correspondence to Yasuhiro Nakazawa (nakazawa@chem.sci.osaka-u.ac.jp) or Takumi Konno (konno@chem.sci.osaka-u.ac.jp).

The structural analysis by the synchrotron X-ray

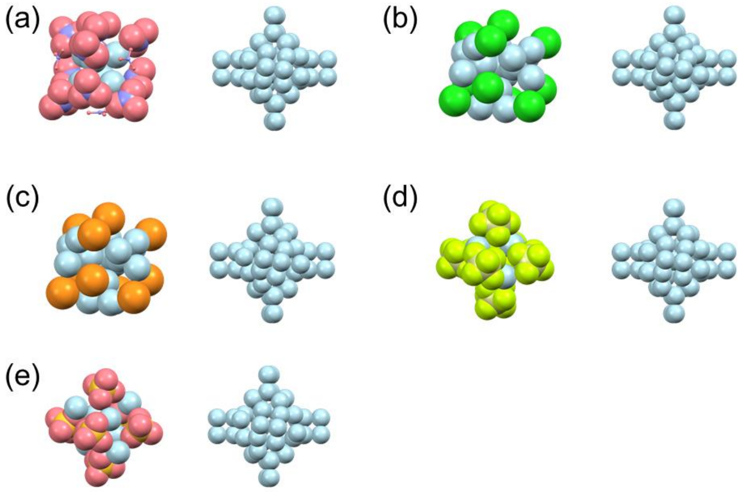

Fig. S1. Structures of inorganic-anion and water clusters in [**1**]$X_2 \cdot nH_2O$ [X_2 = (a) NO_3, (b) Cl_2, (c) Br_2, (d) SiF_6, and (e) SO_4].

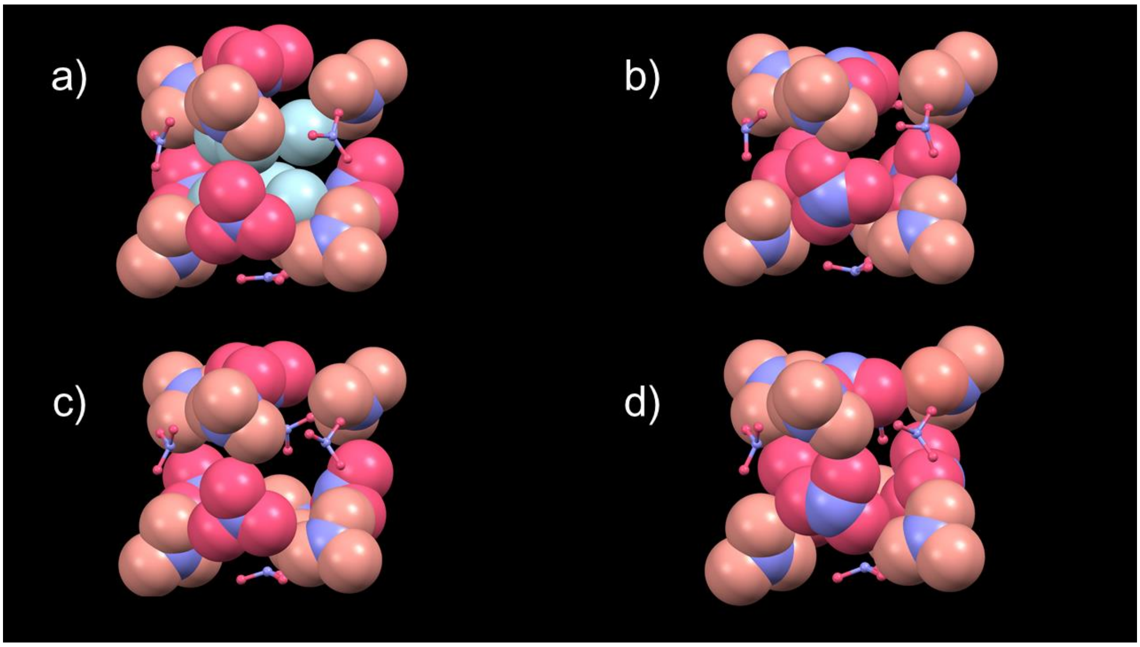

Fig. S2. Structures of anion clusters in [**1**]$(NO_3)_2 \cdot nH_2O$ at (a) 100 K, (b) 300 K, (c) 343 K, and (d) 423 K. The 4 nitrate anions that are disordered into two sets of tetrahedral sites are represented by space filling and ball & stick models with pink color of O atoms.

2. Thermal Analysis

The simultaneous measurement of the differential thermal analysis (DTA) and the thermogravimetric analysis (TGA) of several complexes of CS-NCIS was performed using a commercially available apparatus (Perkin Elmer STA 6000/TG-DTA/DSC) with a constant flow of N_2 50 mL/min. gas atmosphere. The DTA curves of [**1**]$(ClO_4)_2 \cdot nH_2O$ and those of [**1**]$(NO_3)_2 \cdot nH_2O$ with different anion clusters were recorded with a heating rate of 1 K/min, and the results are shown in Figs. S3 (a) and (b) with red squares. The TGA curves are also shown in Figs. S3 (a) and (b) with green circles. The sample weights of the measurements were 7.68 mg and 8.11 mg.

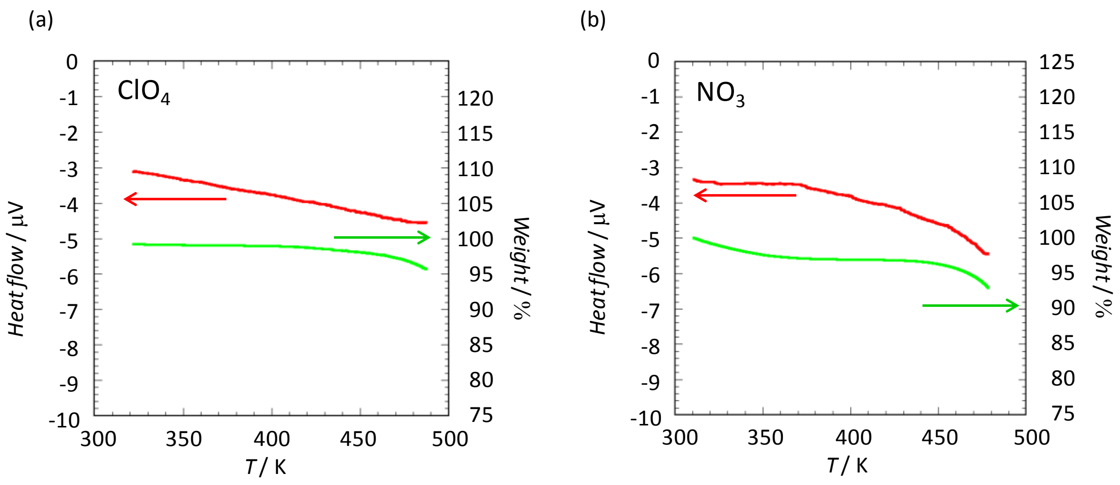

Fig. S3. The DTA and TGA curves obtained for (a) [**1**]$(ClO_4)_2 \cdot nH_2O$ and (b) [**1**]$(NO_3)_2 \cdot nH_2O$

3. The activation energy estimated by the dielectric permittivity measurements

The activation energy related to the dielectric jump was determined from the temperature dependency of the relative permittivity of [**1**](ClO_4)$_2$·$n$$H_2O$, [**1**](Cl)$_2$·$n$$H_2O$, [**1**]($NO_3$)$_2$·$n$$H_2O$ and [**1**](NO_3)$_2$·$n$$D_2O$. Since we could not detect a distinct peak structure in the temperature dependency of $\varepsilon'/\varepsilon_0$, we used the onset temperature of the dielectric jump for the evaluation. The activation plot and determination of the possible activation energy are shown in Fig. S4. Those of other compounds are also in the range of 50-100 kJ $K^{-1}mol^{-1}$, although the estimation of the onset temperatures has some ambiguity of ±10 kJ $K^{-1}mol^{-1}$.

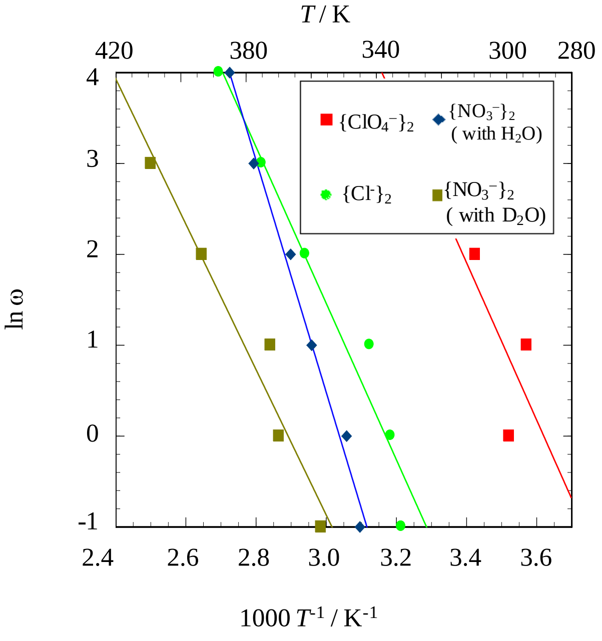

Anion	ΔE / kJ$K^{-1}mol^{-1}$
$\{ClO_4^-\}_2$	7.21 × 10^1
$\{Cl^-\}_2$	7.16 × 10^1
$\{NO_3^-\}_2$ (with H_2O)	1.06 × 10^2
$\{NO_3^-\}_2$ (with D_2O)	6.65 × 10^1

Fig. S4. The relation between the onset temperature of the abrupt jump in dielectric constant and frequency. The activation energies evaluated from this relation are summarized in the table.

4. The effect of deuteration on the dielectric jump

We show in Figs. S5 (a) and (b) that the temperature and frequency dependencies of [**1**](SO_4)·$n$$H_2O$ and [**1**](SiF_6)·$n$$H_2O$, of which the jump temperature is below room temperature. We also show the temperature dependency of the dielectric jump of their deuterated compounds. Although the overall tendencies are similar, those for the deuterated compounds give the dielectric anomaly at lower temperatures. This is also the case for [**1**]NO_3·$n$$H_2O$ and [**1**]Cl·$n$$H_2O$ shown in Fig. 5 (b) and (c).

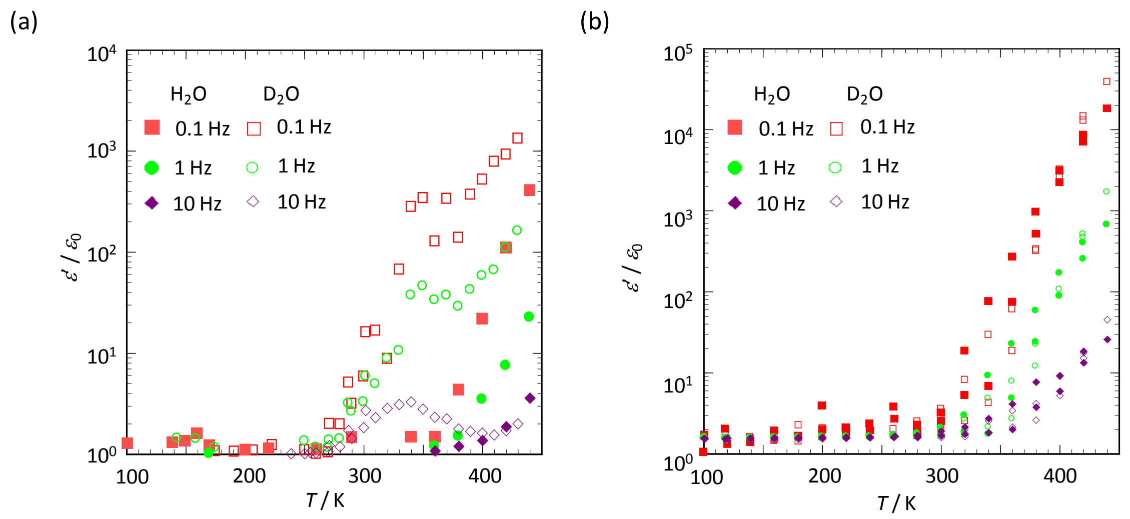

Fig. S5. Temperature dependency of the single crystal of deuterated and pristine NCIS compound with the anion clusters of (a)SO_4^{2-} and (b) SiF_6^{-} obtained at 0.1 Hz, 1.0 Hz, and 10 Hz (closed symbol). The dielectric jump of the deuterated sample (open symbol) is larger than that of the pristine compound.

5. Detection of the electrostriction effect

The electrostriction measurements were performed by using AFM tips with two different configurations, as shown in Fig. S6. The temperature of the ITO plate was controlled using the Pt chip sensor to within ± 0.5 K. The back-ground calibration was performed with epoxy-coated SiO_2 glass with almost the same size of the present compound. The electrostriction was detected in both parallel and perpendicular directions with the applied voltage.

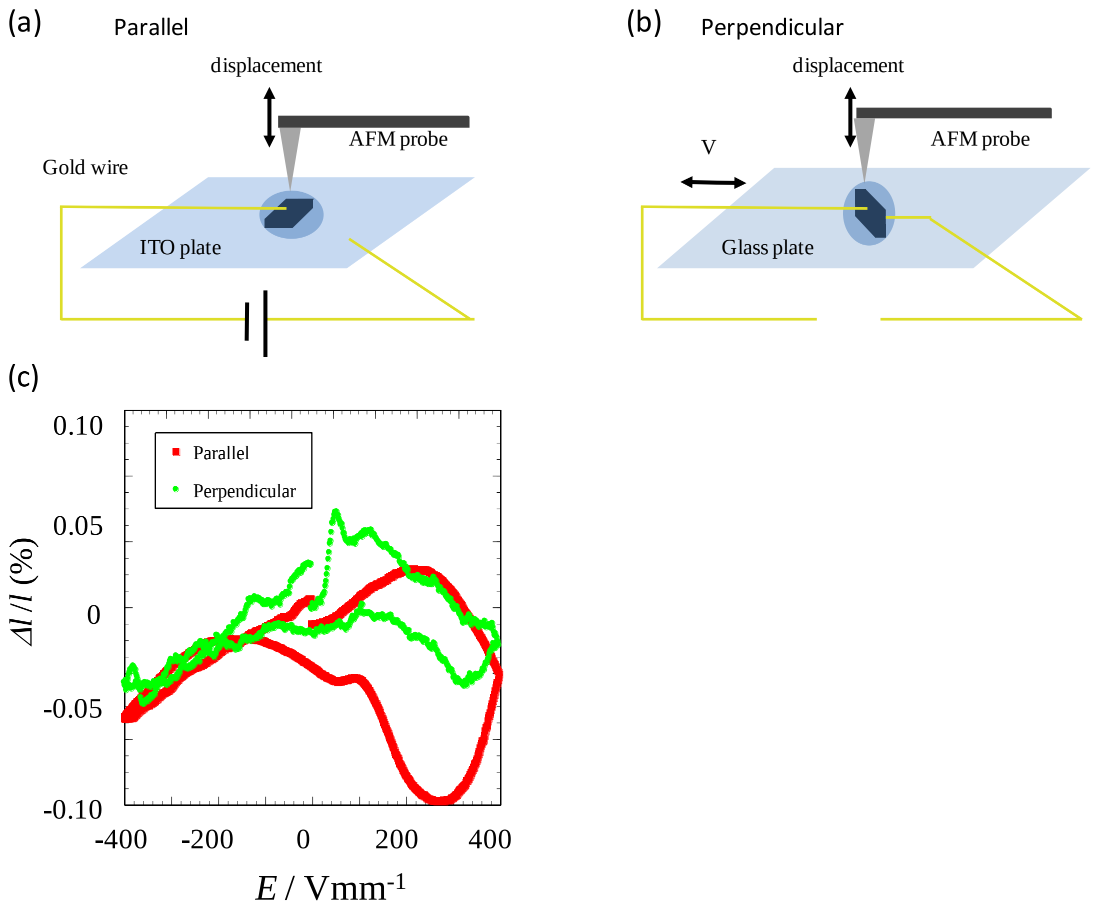

Fig. S6. The schematic illustrations of the setup of the crystal displacement measurements for (a) parallel and (b) perpendicular directions. (c) The displacement of the sample occurs by sweeping the electric field between -400 Vmm^{-1} and 400 V mm^{-1} measured in the parallel and perpendicular configurations of the directions of displacement measurements and applied DC voltage.

The crystal structure analysis under applied electric fields was performed by the special set-up in the X-ray Syncrotron radiation port. The two lead wires were attached to a single piece of crystal of [**1**]Cl·$n$$H_2O$, and the sample was coated with Epoxy. The decrease of the lattice parameters upon applying an electric field was confirmed by tracing the shift of Bragg spot with increasing electric fields, as shown in Fig. S7.

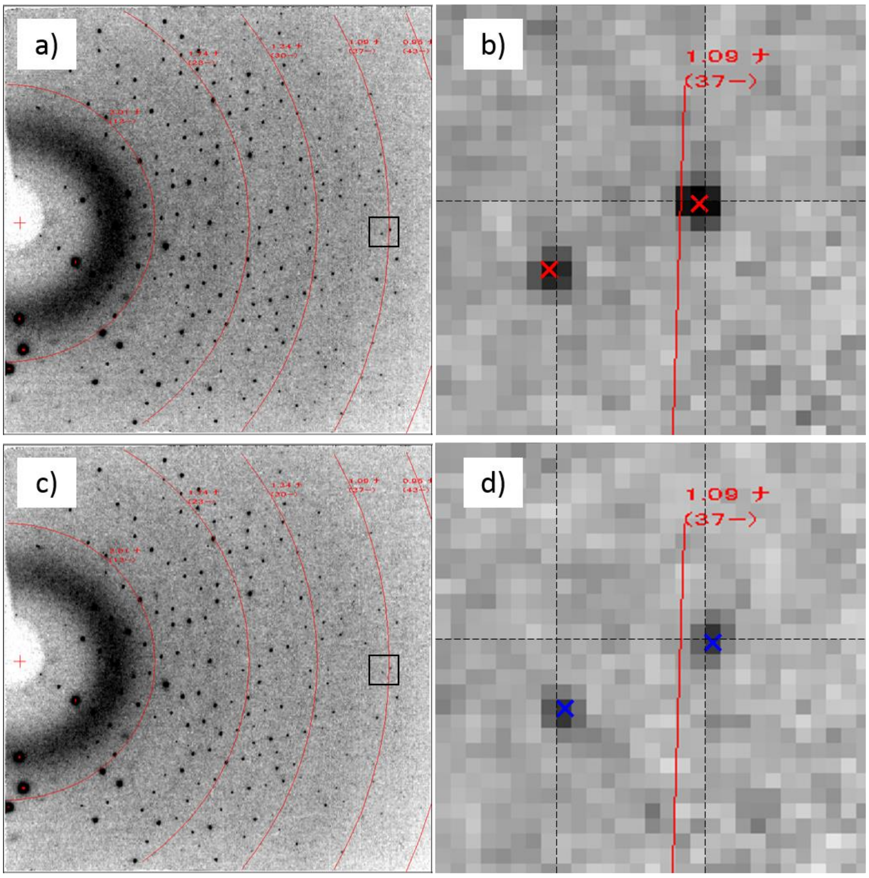

Fig. S7. X-ray diffraction images for the tracing of the positions of the Bragg spots with increasing DC voltage. a) The diffraction image collected at 380 K with 0 V. b) The enlarged figure of the square in a). c) The diffraction image collected at 380 K with 100 V. d) The enlarged figure of the square in c).

6. Details of quantum mechanics (QM)/molecular mechanics (MM) MD simulations

The model we employed for QM/MM MD calculations is illustrated in Fig. S8. The QM part consists of a Cl cluster ($\{Cl^-\}_{10}$), 12 water molecules in the cavity that is located around the centre in Fig. S8, and 4 water molecules at the tetrahedral edges of the cavity. The MM part consists of 12 $[Au^I_4Co^{III}_2(dppe)_2(\text{D-pen})_4]^{2+}$ complexes surrounding the previous model, and 24 water molecules that are sandwiched between the complexes. The initial positions of the heavy atoms in this model were taken from the X-ray structure of the ionic solid, $[Au^I_4Co^{III}_2(dppe)_2(\text{D-pen})_4]Cl_2{\cdot}n(H_2O)$, and the hydrogen atoms of all the water molecules were added using UCSF Chimera[20]. The general AMBER force field (GAFF)[21] was used for all organic parts of the complex, in which the effective point charges were replaced by the electrostatic potential (ESP) charges [22] that were obtained from ab initio B3LYP/LANL2DZ calculations for the complex. For Co and Au ions, we employed Lennard-Jones (LJ) potentials, which are those based on the Pauling ionic radii[2] and the LJ parameters taken from previous research. [23-25] Since our purpose was to see the behaviour of the Cl cluster with water molecules in the cavity, we fixed the framework consisting of the 12 complexes and the positions of the oxygens of 24 water molecules intercalated between the complexes. The Cl cluster ($\{Cl^-\}_{10}$) and all water molecules, which are shown in the stick model in Fig. S8, are treated at the quantum mechanical (QM) level. In this study, we employed the semi-empirical PM3-MAIS method for the QM level, which is designed to reproduce hydrogen bonds and other interactions of $HCl\text{-}(H_2O)_n$ clusters. [26]

The QM/MM MD simulation was performed with Amber14[27] in the following steps: (i) the protons of the water molecules were relaxed with 100 steps at T = 0 K, (ii) the temperature was increased up to T = 300 K for 100 ps, (iii) the equilibration run was performed for 100 ps, and finally (iv) the production run was performed for 1 ns at T = 300 K. Except for the first relaxation step, all runs were simulated in the NVT ensemble with a time step of 1 fs in the Berendsen's thermostat. [28]

We estimated the probability density of each atom during the QM/MM MD trajectory using the ensemble analysis tool of Chimera.[29] This tool counts how many times a specified atom falls within a grid cell; we set the resolution of the grid cell to be 0.25 Å. The probability density values described in the text were obtained simply by dividing the counts by the number of steps examined (for details, see the Chimera's user guide[31]).

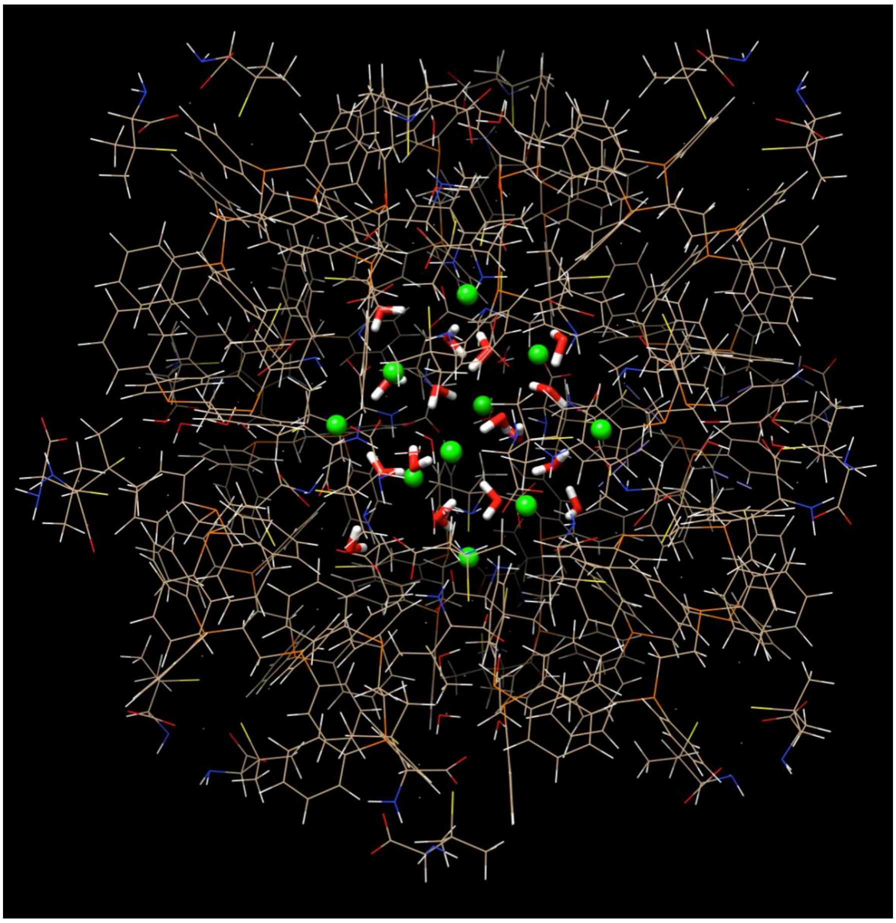

Fig. S8 The QM/MM model of $[Au^{I}_{4}Co^{III}_{2}(dppe)_{2}(\text{D-pen})_{4}]Cl_{2}\cdot nH_{2}O$. The atoms of the QM region are presented in stick mode, while those of the MM region in the wire model.

7. Comparison between the QM/MM MD and ab initio QM results

To compare the most stable structure obtained with ab initio QM calculation (at T = 0 K) and the probability density of the cluster obtained with the QM/MM MD calculation (at T = 300 K), we also performed a B3LYP/6-31G** calculation with using Gaussian09 rev. C.[30] Although we tried to optimize the geometry of $(Cl^-)_{10}$-$(H_2O)_{12}$ in the cavity under the electrostatic field caused by the other molecules, the optimization procedure was not completed due to numerical errors. Instead, we show the stable structure after 72 steps of the optimization procedure, together with the equivalued surfaces of probability densities ($r(X) = 0.005$, X = Cl^-, O, H), in Fig. S9. From this figure, we found that the stable structure obtained with ab initio calculation is considerably different from that obtained with the QM/MM MD calculation. In particular, the $\{Cl^-\}_{10}$ anion cluster expanded in comparison with that of the X-ray structure, implying that the X-ray positions of the anion cluster with water molecules lie in some metastable points, which are well reproduced by the QM/MM MD calculations at finite temperature. We explain the quantum mechanics (QM)/molecular mechanics(MM) MD simulations procedure used in this work in detail.

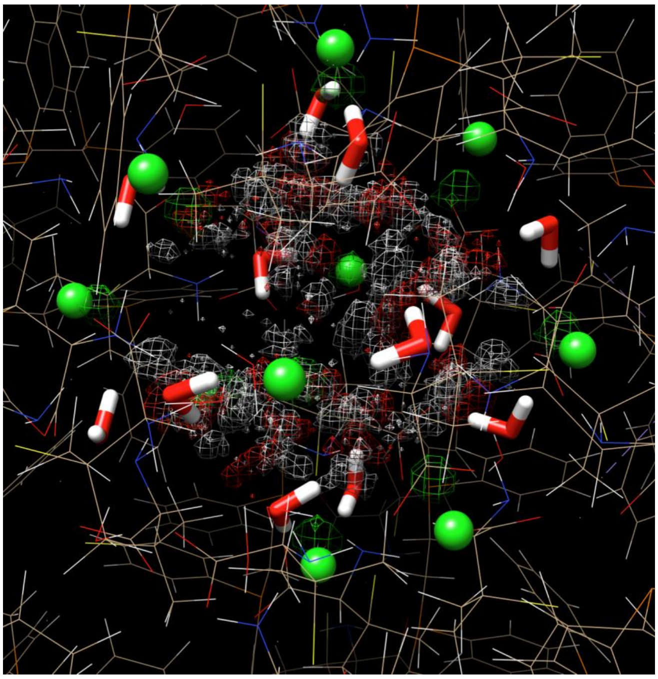

Fig. S9. The stable structure of $(Cl^{-})_{10}$-$(H_2O)_{12}$ obtained with the ab initio QM method (for details, see the text). The Cl ions shown as the green spheres and water molecules in the stick model are from the calculation results. The wire meshes are the isosurfaces of the probability densities of Cl^{-}, O, and H atoms ($r(X) = 0.005$, X = Cl^{-}, O, H), which were obtained from the QM/MM MD calculations shown above.

References for Supplemental Information

20. Pettersen, E.F., Goddard, T.D., Huang, C.C., Couch, G.S., Greenblatt, D.M., Meng, E.C., and Ferrin, T.E. UCSF Chimera - A Visualization System for Exploratory Research and Analysis. *J. Comput. Chem.* **25**, 1605-1612 (2004).
21. Wang, J., Wolf, R. M., Caldwell, J. W., Kollman, P. A., & Case, D. A. "Development and testing of a general AMBER force field". *J. Comp. Chem.*, **25**, 1157-1174 (2004).
22. Besler, B. H., Merz Jr., K. M., & Kollman, P. A., Atomic charges derived from semiempirical methods, *J. Comp. Chem.*, **11**, 431-439 (1990).
23. Pu, Q., Leng, Y., Zhao, X., & Cummings, P.T., Molecular simulations of stretching gold nanowires in solvents, Nanotechnology **18**, 424007 (1-7), (2007).
24. Li, P.; Roberts, B. P.; Chakravorty, D. K.; Merz, K. M., Jr Rational Design of Particle Mesh Ewald Compatible Lennard-Jones Parameters for + 2 Metal Cations in Explicit Solvent. J. Chem. Theor. Comput. **9**, 2733-2748 (2013).
25. Li, P., Merz Jr. K. M. Metal Ion Modeling Using Classical Mechanics, Chem. Rev. **117**, 1564-1686 (2017).
26. Arillo-Flores, O. I., Ruiz-Lopez, M. F., Bernal-Uruchurtu, M. I., Can semi-empirical models descryibe HCl dissociation in water? *Theor. Chem. Acc.* **118**, 425-435 (2007).
27. Case, D.A., Babin, V., Berryman, J.T., Betz, R.M., Cai, Q., Cerutti, D.S., Cheatham III, T.E., Darden, T.A., Duke, R.E., Gohlke, H., Goetz, A.W., Gusarov, S., Homeyer, N., Janowski, P., Kaus, J., Kolossváry, I., Kovalenko, A., Lee, T.S., LeGrand, S., Luchko, T., Luo, R., Madej, B.. Merz, K.M., Paesani, F., Roe, D.R., Roitberg, A., Sagui, C., Salomon-Ferrer, R., Seabra, G., Simmerling, C.L., Smith, W., Swails, J., Walker, R.C., Wang, J., Wolf, R.M. Wu X., & P.A. Kollman (2014), AMBER 14, University of California, San Francisco.
28. Berendsen, H. J. C., Postma, J. P. M., van Gunsteren, W.F., DiNola, A., & Haak, J.R. Molecular dynamics with coupling to an external bath, *J. Chem. Phys.*, **81**, 3684–3690 (1984).
29. https://www.cgl.ucsf.edu/chimera/docs/ContributedSoftware/movie/framemovie.html
30. Gaussian 09, Revision C.01, Frisch, M. J., Trucks, G. W., Schlegel, H. B., Scuseria, G. E., Robb, M. A., Cheeseman, J. R., Scalmani, G., Barone, V., Mennucci, B., Petersson, G. A., Nakatsuji, H., Caricato, M., Li, X., Hratchian, H. P., Izmaylov, A. F., Bloino, J., Zheng, G., Sonnenberg, J. L., Hada, M., Ehara, M., Toyota, K., Fukuda, R., Hasegawa, J., Ishida, M., Nakajima, T., Honda, Y., Kitao, O., Nakai, H., Vreven, T., Montgomery, Jr., J. A., Peralta, J. E., Ogliaro, F., Bearpark, M., Heyd, J. J.. Brothers, E., Kudin, K. N., Staroverov, V. N., Keith, T., Kobayashi, R., Normand, J., Raghavachari, K., Rendell, A., Burant, J. C., Iyengar, S. S., Tomasi, J., Cossi, M., Rega, N., Millam, J. M., Klene, M., Knox, J. E., Cross, J. B., Bakken, V., Adamo, C., Jaramillo, J., Gomperts, R., Stratmann, R. E., Yazyev, O., Austin, A. J., Cammi, R., Pomelli, C., Ochterski, J.

W., Martin, R. L., Morokuma, K., Zakrzewski, V. G., Voth, G. A., Salvador, P., Dannenberg, J. J., Dapprich, S., Daniels, A. D., Farkas, O., Foresman, J. B., Ortiz, J. V., Cioslowski, J., Fox, D. J., Gaussian, Inc., Wallingford CT, 2010.
